# The complete mitogenome of *Glyptothorax deqinensis* (Siluriformes: Sisoridae)

**DOI:** 10.1080/23802359.2019.1664950

**Published:** 2019-09-23

**Authors:** Tingbing Zhu, Chuankun Zhu, Yongfeng He, Deguo Yang

**Affiliations:** a Key Laboratory of Freshwater Biodiversity Conservation, Ministry of Agriculture and Rural Affairs of China, Yangtze River Fisheries Research Institute, Chinese Academy of Fishery Sciences, Wuhan, Hubei, China;; b Jiangsu Engineering Laboratory for Breeding of Special Aquatic Organisms, Huaiyin Normal University, Huai’an, Jiangsu, China

**Keywords:** *Glyptothorax deqinensis*, mitogenome, Sisoridae

## Abstract

*Glyptothorax deqinensis* is a small endangered Sisoridae fish mainly distributed in the southwest of China. In the present study, the complete mitochondrial genome of *G. deqinensis* was firstly sequenced. The total length of the *G. deqinensis* mitogenome is 16,542 bp, including 13 protein-coding genes, two ribosomal RNA genes, 22 transfer RNA genes, and a control region. Among these genes, 28 are encoded by the H-strand and 9 by the L-strand. The information mitogenome obtained herein will provide useful tools for future studies on population genetic and phylogenetic analyses of this fish.

*Glyptothorax deqinensis* is a small benthic Sisoridae fish which naturally distributed in the southwest of China (Chu and Chen 1990). Because of influences of human activities, such as overfishing, water pollution, habitat destruction, and water conservancy project construction (Shao et al. 2016), natural resources of *G. deqinensis* has sharply declined in recent years, and it has been listed as endangered fish species in China (Jiang et al. 2016). However, studies on this fish are quite limited, and no available genetic information has been reported presently. In this study, the complete mitochondrial genome (mtgenome) of *G. deqinensis* was sequenced and characterized for the first time.

Sample of *G. deqinensis* was collected from the Deqin (Yunnan province, China) section of the Lancang River (N 28°32′27.15″, E 98°47′52.53″). Fin clips of samples were taken and stored in anhydrous ethanol at 4 °C. After sampling, the specimen was stored in 95% ethanol and kept in the herbarium of Yangtze River Fisheries Research Institute (accession number LC20180411002). Total DNA was extracted following the standard phenol/chloroform method (Sambrook and Russell 2001), and DNA quality was measured by a Nanodrop2000 spectrophotometer (Thermo Scientific, USA). Using the mitogenome of *Glyptothorax trilineatus* (NC_021608.1) as a reference sequence, 18 pairs of primers were designed to amplify mitochondrial DNA segments of *G. deqinensis*. Genome annotation was performed using MITOS2 webserver (Bernt et al. 2013), and compositions of the four nucleotides were calculated using MEGA4 (Tamura et al. 2007).

After sequencing and assembly of mtDNA segments amplified by the 18 primer pairs, a circular mtgenome with a length of 16,542 bp was finally obtained (GenBank accession no. MN082046), which was similar to that of other fishes from the genus *Glyptothorax* (Li 2016; Huang et al. 2017). The overall nucleotide composition for A, T, G, and C is 31.2, 25.2, 15.5, and 27.1%, respectively, with a G + C content of 42.6%. After annotations of the *G. deqinensis* mitogenome, 37 genes, including 13 protein-coding genes, two ribosomal RNA genes (12S rRNA and 16S rRNA) and 22 transfer RNA genes, were identified and a putative control region was also found. Among the 37 genes, 28 genes are encoded by the heavy (H) strand, whereas the other 9 genes, including a protein-coding gene (*ND6*) and 8 tRNA genes (*tRNA^Gln^*, *tRNA^Ala^*, *tRNA^Asn^*, *tRNA^Cys^*, *tRNA^Tyr^*, *tRNA^Ser^*, *tRNA^Glu^*, and *tRNA^Pro^*) are located on the light (L) strand. The mtgenomic structure of *G. deqinensis* was the same as that of other fishes from *Glyptothorax* (Li 2016; Huang et al. 2017).

Among the 13 protein-coding genes, 12 genes used typical metazoan start codon ATG, while the start codon was GTG for *COXI*. Termination codon for 6 of the 13 protein-coding genes were normal, with TAA for *ND1*, *COI*, *ATPase8*, *ND4L*, and *ND5*, and TAG for *ND6*, while the remaining 7 genes ended with incomplete stop codon of T- (*ND2*, *COXII*, *COXIII*, *ND3*, *ND4*, and *Cytb*) and TA- (*ATPase 6*).

Moreover, mtDNA sequences of 10 Sisoridae fishes were downloaded from GenBank for phylogenetic analysis. The 10 sequences were from *Glyptothorax cavia* (KY230517.1), *Glyptothorax fokiensis* (JQ917224.1), *Glyptothorax sinensis* (KJ739617.1), *Glyptothorax zanaensis* (KU212205.1), *Glyptothorax trilineatus* (JQ026262.1), *Glyptothorax laosensis* (KP271438.1), *Gagata dolichonema* (JQ026250.1), *Pseudecheneis sulcata* (JQ026259.1), *Glaridoglanis andersonii* (JQ026254.1), and *Glyptosternon maculatum* (JQ026251.1). The neighbor-joining (N-J) method in MEGA 4.0 (Tamura et al. 2007) was used to perform phylogenetic analyses. The N-J tree showed that *G. deqinensis* was clustered together with *Glyptothorax* fishes under strong bootstrap value support (Figure 1), which confirmed the phylogenetic status of *G. deqinensis*, and provided useful information for further phylogenetic and evolutionary studies on the genus *Glyptothorax*, as well as the family Sisoridae.

**Figure 1.**
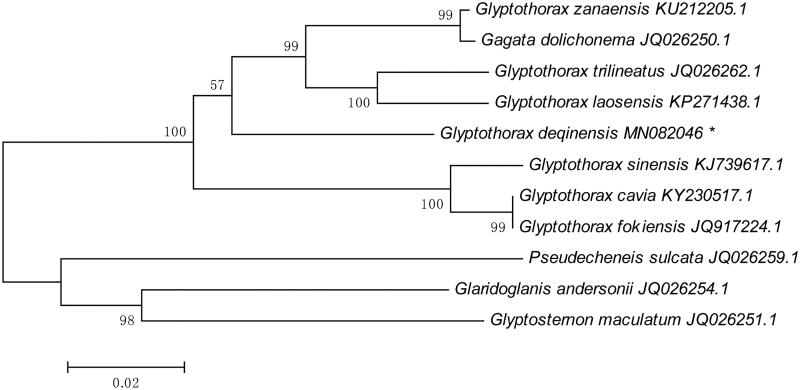
The neighbor-joining phylogenetic tree of *Glyptothorax deqinensis* with other Sisoridae species based on mitochondrial genome sequences. Numbers along with branches are Bayesian posterior probability and bootstrap values for the phylogenetic tree.

## References

[CIT0001] Bernt M, Donath A, Juhling F, Externbrink F, Florentz C, Fritzsch G, Putz J, Middendorf M, Stadler PF 2013 MITOS: improved de novo metazoan mitochondrial genome annotation. Mol Phylogenet Evol. 69:313–319. 22982435 10.1016/j.ympev.2012.08.023

[CIT0002] Chu X, Chen Y 1990 The fishes of Yunnan part II. Beijing (China): Science Press.

[CIT0003] Huang F, Liu M, Yu L, Liu S 2017 The complete mitochondrial genome sequence of *Glyptothorax laosensis* (Siluriformes, Sisoridae). Mitochondrial DNA Part A. 28:60–61. 10.3109/19401736.2015.1110798 26710121

[CIT0004] Jiang Z, Jiang J, Wang Y, Zhang E, Zhang Y, Li L, Xie F, Cai B, Cao L, Zheng G, et al. 2016 Red list of China’s vertebrates. Biodiv Sci. 24:500–551.

[CIT0005] Li B 2016 The complete mitochondrial genome of *Glyptothorax cavia* (Siluriformes, Sisoridae, Glyptothorax): genome characterization and phylogenetic analysis. Mitochondrial DNA Part B. 2:259–260. 10.1080/23802359.2017.1307701 PMC7800425 33473791

[CIT0006] Sambrook J, Russell DW 2001 Molecular cloning: a laboratory manual (3rd ed.). New York (NY): Cold Spring Harbor Laboratory Press.

[CIT0007] Shao K, Yan S, Zhu B, Xu N, Li W, Xiong M 2016 Complete mitochondrial genome of *Pareuchiloglanis sinensis* (Siluriformes: Sisoridae). Mitochondrial DNA A: DNA Mapp Seq Anal. 27:713–714. 24801372 10.3109/19401736.2014.913155

[CIT0008] Tamura K, Dudley J, Nei M, Kumar S 2007 MEGA4: Molecular Evolutionary Genetics Analysis (MEGA) software version 4.0. Mol Biol Evol. 24:1596–1599. 17488738 10.1093/molbev/msm092

